# Supplementary material for: Microrobot collectives with reconfigurable morphologies, behaviors, and functions
Source: Nat Commun. 2022 Apr 26;13:2239. doi: 10.1038/s41467-022-29882-5 (PMC9043221; doi:10.1038/s41467-022-29882-5)
Supplement: Supplementary file 1 — Supplementary Information [file 41467_2022_29882_MOESM1_ESM.pdf]

Supplementary Information for

Microrobot Collectives with Reconfigurable Morphologies, Behaviors, and Functions

The PDF file includes:

Supplementary Fig. 1. Experimental setup.
Supplementary Fig. 2. Additional collective behavior analysis.
Supplementary Fig. 3. Characterization of simulated rotating collectives with 120 micro-disks.
Supplementary Fig. 4. Global hexatic order parameter for 126 micro-disks in rotating, static, and oscillating modes.
Supplementary Fig. 5. Experimental characterization of 34 micro-disks in rotating, oscillating, and static modes.
Supplementary Fig. 6. Velocity characterization of collectives with 7, 34, and 60 micro-disks exhibiting the GaSPP mode.
Supplementary Fig. 7. Chain locomotion speeds for different frequencies when there are x and y gradients of 0.7 Gauss/mm.
Supplementary Fig. 8. Locomotion speeds across various gradients and frequencies for chains aligned along the y direction.
Supplementary Fig. 9. Flow-induced collective object manipulation in the rotation mode.
Supplementary Fig. 10. Collective radius when the micro-disks are within ring-like structures packed at various area fractions.
Supplementary Fig. 11. Mean angular velocity of micro-disks when they are within a ring-like structure.
Supplementary Fig. 12. Collective separates into two clusters by using the arena boundary and then reforms a single cluster.

Other Supplementary Material for this manuscript includes the following:

Supplementary Movie 1. Overview of different collective formations.
Supplementary Movie 2. First sequence of transitions between collective formations.
Supplementary Movie 3. Simulations of transitions between collective formations.
Supplementary Movie 4. Second sequence of transitions between collective formations.
Supplementary Movie 5. Third sequence of transitions between collective formations.
Supplementary Movie 6. Coordinated locomotion of the collective in different formations.
Supplementary Movie 7. Magnetic field gradient control for “MPI” and “C” trajectories.
Supplementary Movie 8. Collective navigation through an intricate environment.
Supplementary Movie 9. Contact-based object transport using magnetic field gradients.
Supplementary Movie 10. Visualization of flow around the rotating collective.
Supplementary Movie 11. Flow-induced object transport using the rotating collective.
Supplementary Movie 12. Ring rotation from inside and outside.
Supplementary Movie 13. Torque transfer between two rings.
Supplementary Movie 14. Rotation of various shapes.
Supplementary Movie 15. Comparison of dispersion using the rotation and GaSPP modes.
Supplementary Movie 16. Collective splitting using the rotation mode.
Supplementary Movie 17. Collective splitting using the GaSPP mode.

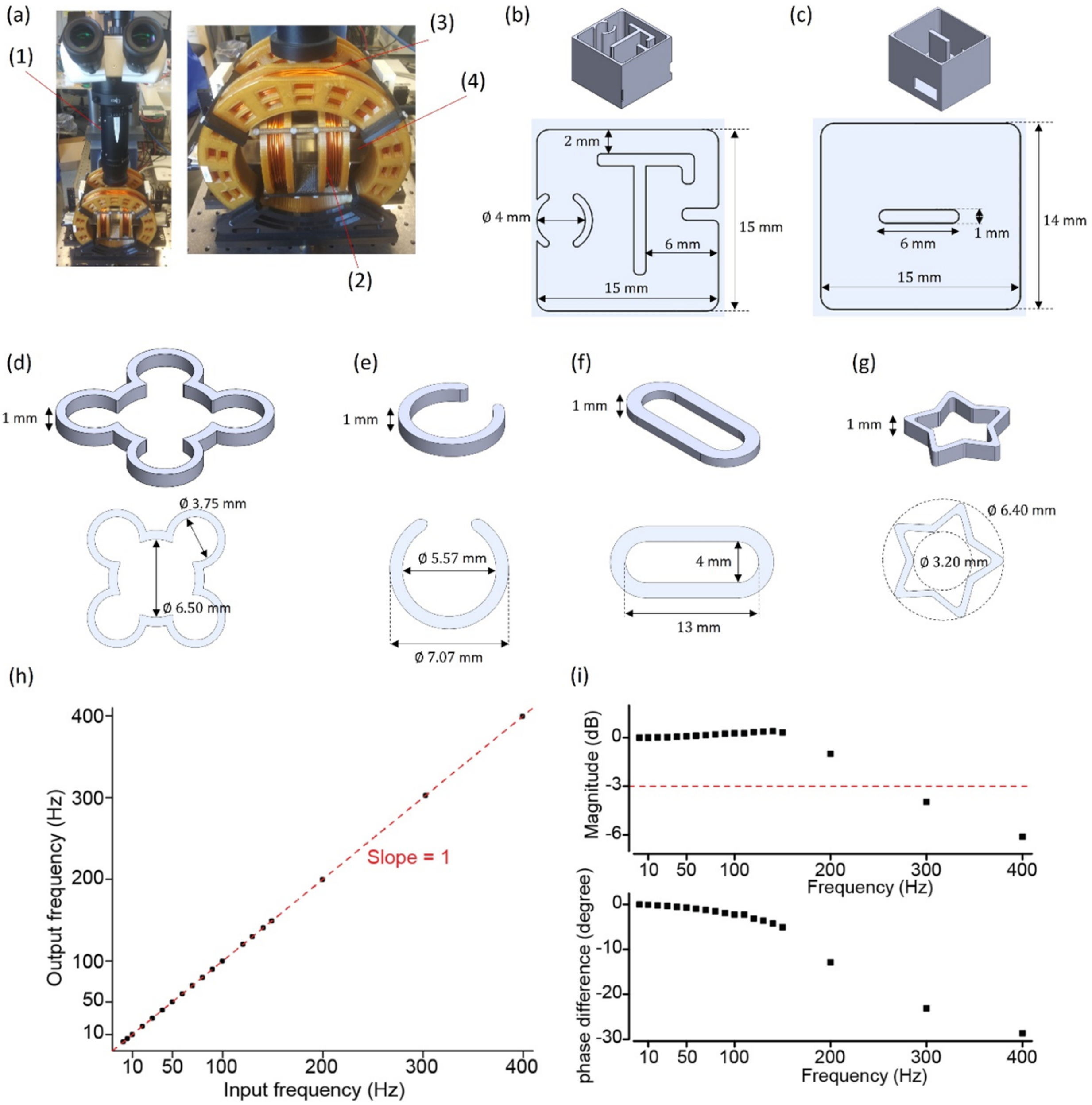

Supplementary Fig. 1. Experimental setup. (**a**) Two-axis Helmholtz coils and imaging setup: (**1**) Manual and computer view onto stage; (**2**) X-coils; (**3**) Y-coils; (**4**) Stage. (**b-g**) Arena designs for experimental demonstrations: (b) Maze arena. (c) Ball Motion Arena. (d) Quadrotor object. (e) C-shape object. (f) Rod-like object. (g) Star-like object. (h) Plot of frequency of output coil current vs input signal to generate the magnetic field. The output current has the same frequency as the input signal. (i) Bode plot of magnitude and phase of the coil current. The magnitude of the output current falls below the 3dB line after 200 Hz.

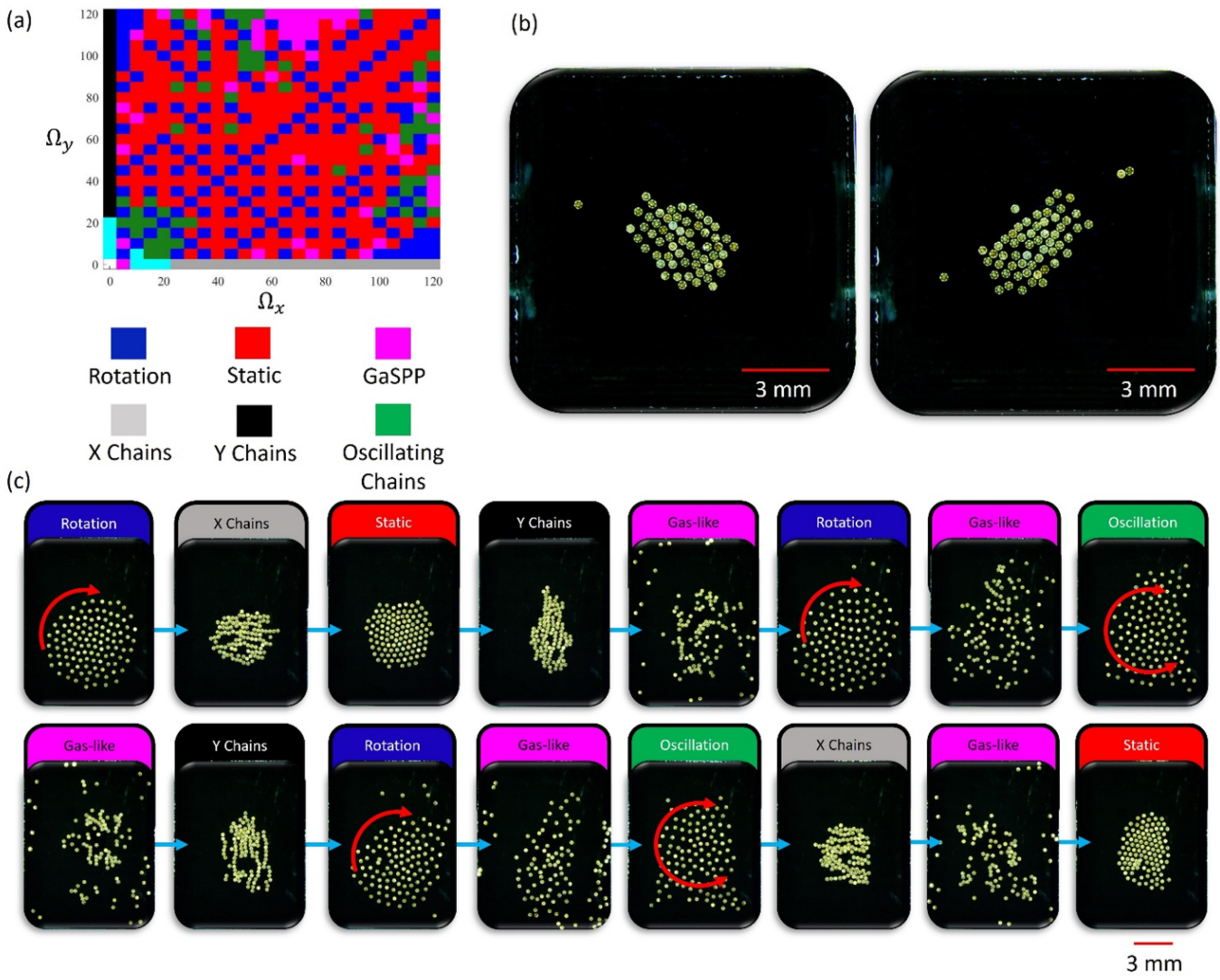

Supplementary Fig. 2. Additional collective behavior analysis. (**a**) State diagram across Ω_x, Ω_y space at 5 Hz increments. Ω_x, Ω_y are the oscillation frequencies of the external magnetic field along *x* and *y* axes respectively. (**b**) Chain formation at two orientations not along the magnetic frequency axes ($\Omega_x = 40$ Hz, $\Omega_y = 20$ Hz: (left) 45° below the *x* axis, (right) 45° above the *x* axis. (**c**) Mode Transitions: (Top) Rotation ($\Omega_{x,y} = 40$ Hz); X-Chains ($\Omega_x = 30$ Hz); Static ($\Omega_x = 60$ Hz); Y-Chains ($\Omega_y = 30$ Hz); GaSPP ($\Omega_y = 70$ Hz); Rotation ($\Omega_{x,y} = 50$ Hz); GaSPP ($\Omega_y = 70$ Hz); Oscillation ($\Omega_y = 40$ Hz). (Bottom) GaSPP ($\Omega_y = 70$ Hz); Y-Chains ($\Omega_y = 30$ Hz); Rotation ($\Omega_{x,y} = 40$ Hz); GaSPP ($\Omega_y = 30$ Hz); Oscillation ($\Omega_y = 30$ Hz); X-Chain ($\Omega_x = 50$ Hz); GaSPP ($\Omega_y = 70$ Hz); Static ($\Omega_x = 140$ Hz).

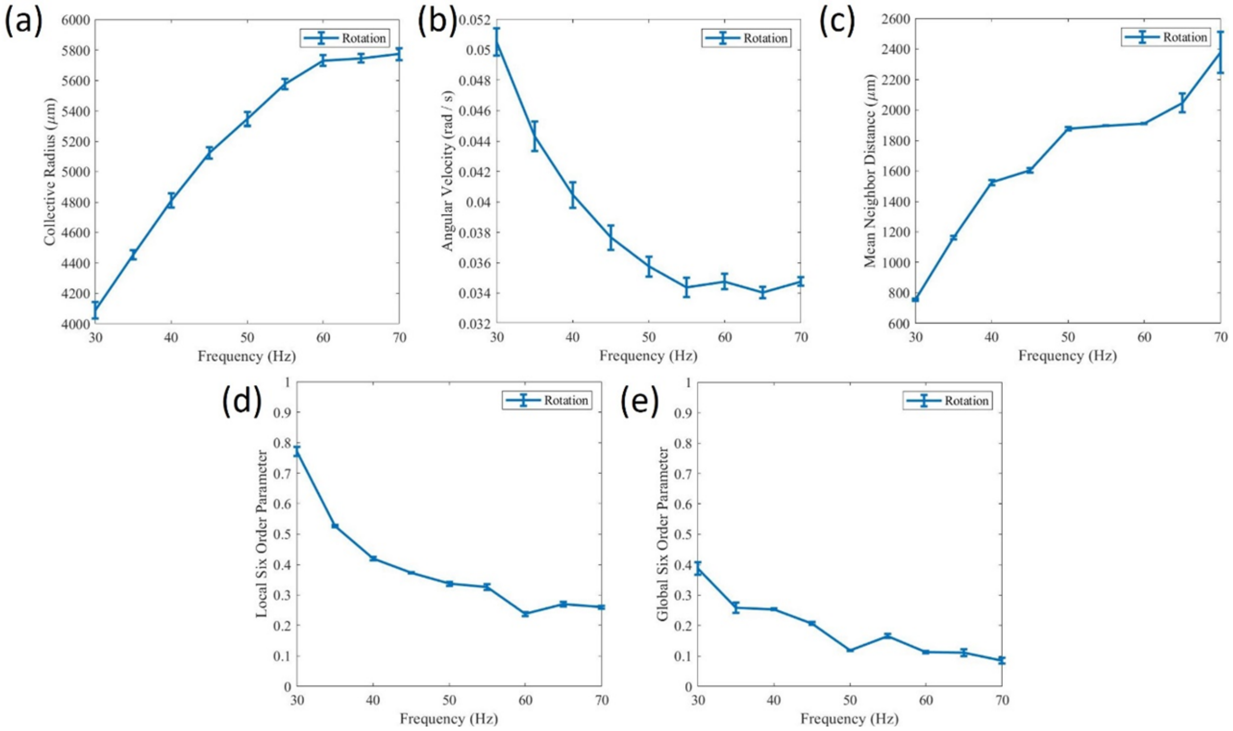

Supplementary Fig. 3. Characterization of the simulated rotating collectives with 120 micro-disks. (**a**) Collectiveradius; (**b**) mean angular velocity; (**c**) mean neighbor distance; (**d**) local hexatic order; (**e**) global hexatic order. The error bars represent standard deviation over 10 s.

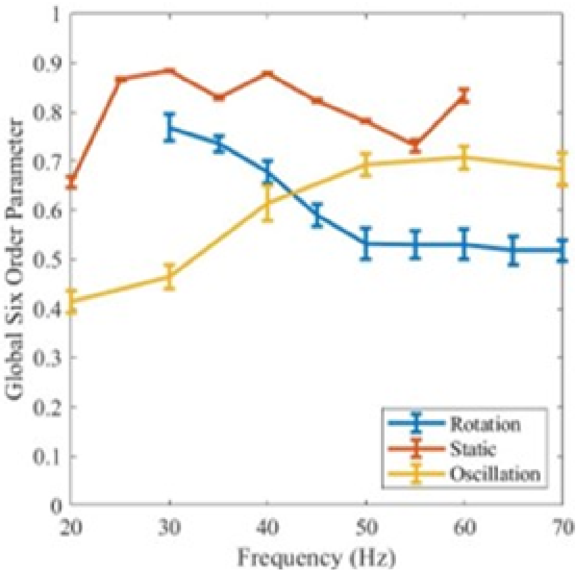

Supplementary Fig. 4. Global hexatic order parameter for 126 micro-disks in the rotating, static, and oscillating modes. The error bars represent standard deviation over 10 s.

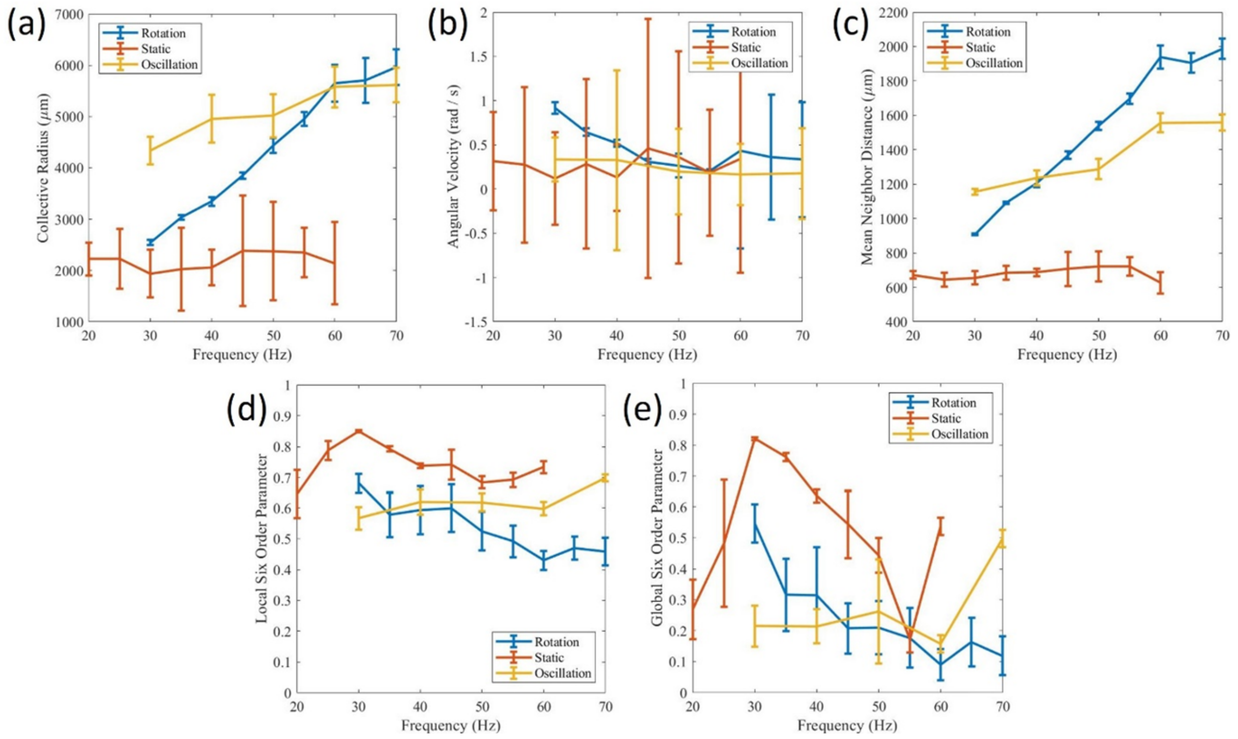

Supplementary Fig. 5. Experimental characterization of 34 micro-disks in the rotating, oscillating, and static modes. (**a**)Collective radius; (**b**) mean angular velocity; (**c**) mean neighbor distance; (**d**) local hexatic order parameter; (**e**) global hexatic order parameter. The error bars represent standard deviation over 10 s.

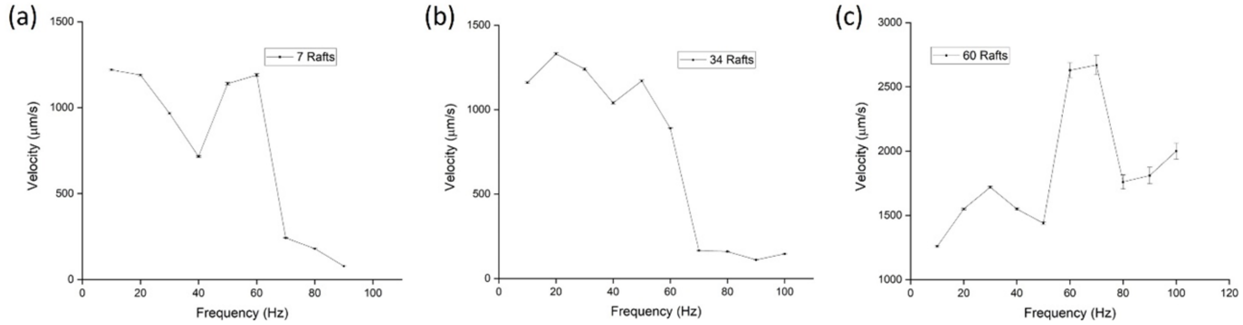

Supplementary Fig. 6. Velocity characterization of collectives with 7, 34, and 60 micro-disks exhibiting the GaSPP mode. Mean velocity of collective with (**a**) 7, (**b**) 34 and (**c**) 60 micro-disks, respectively. The error bars represent standard deviation over 10 s.

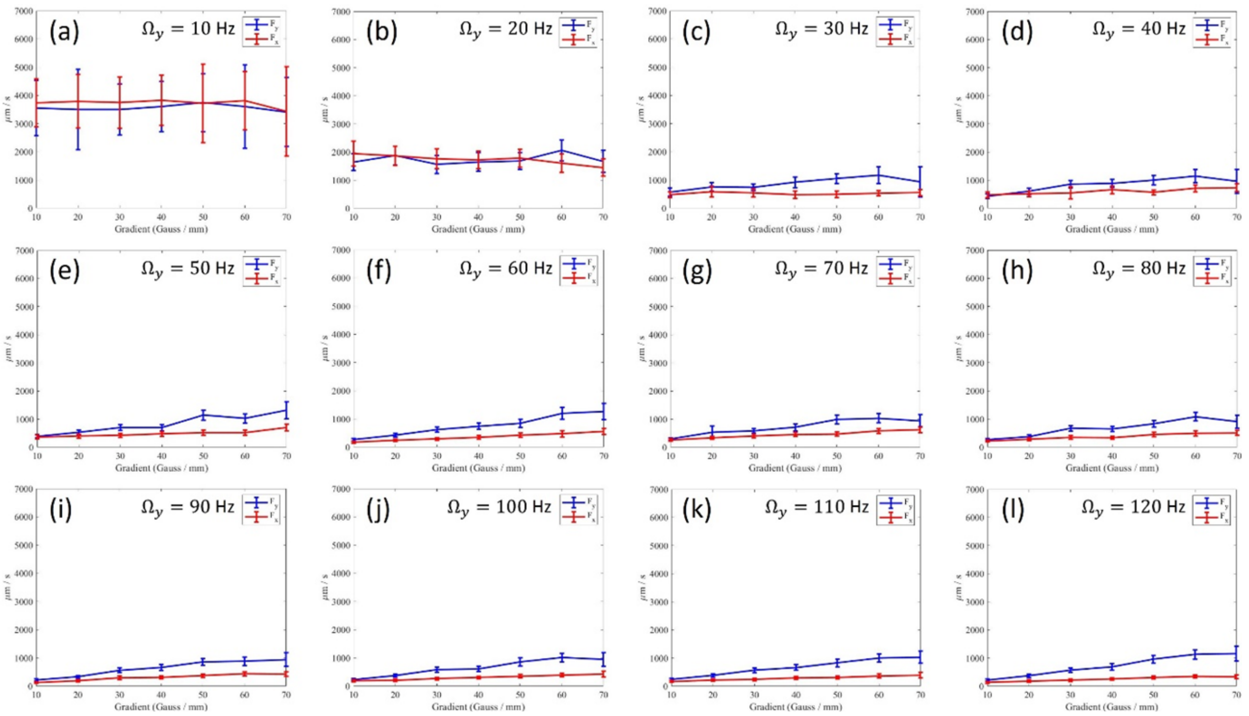

Supplementary Fig. 7. Chain locomotion speeds for different frequencies when there are *x* and *y* gradientsof 0.7 Gauss/mm. (**a-l**) 10-120 Hz at 10 Hz increments. The error bars represent standard deviation over 5 s.

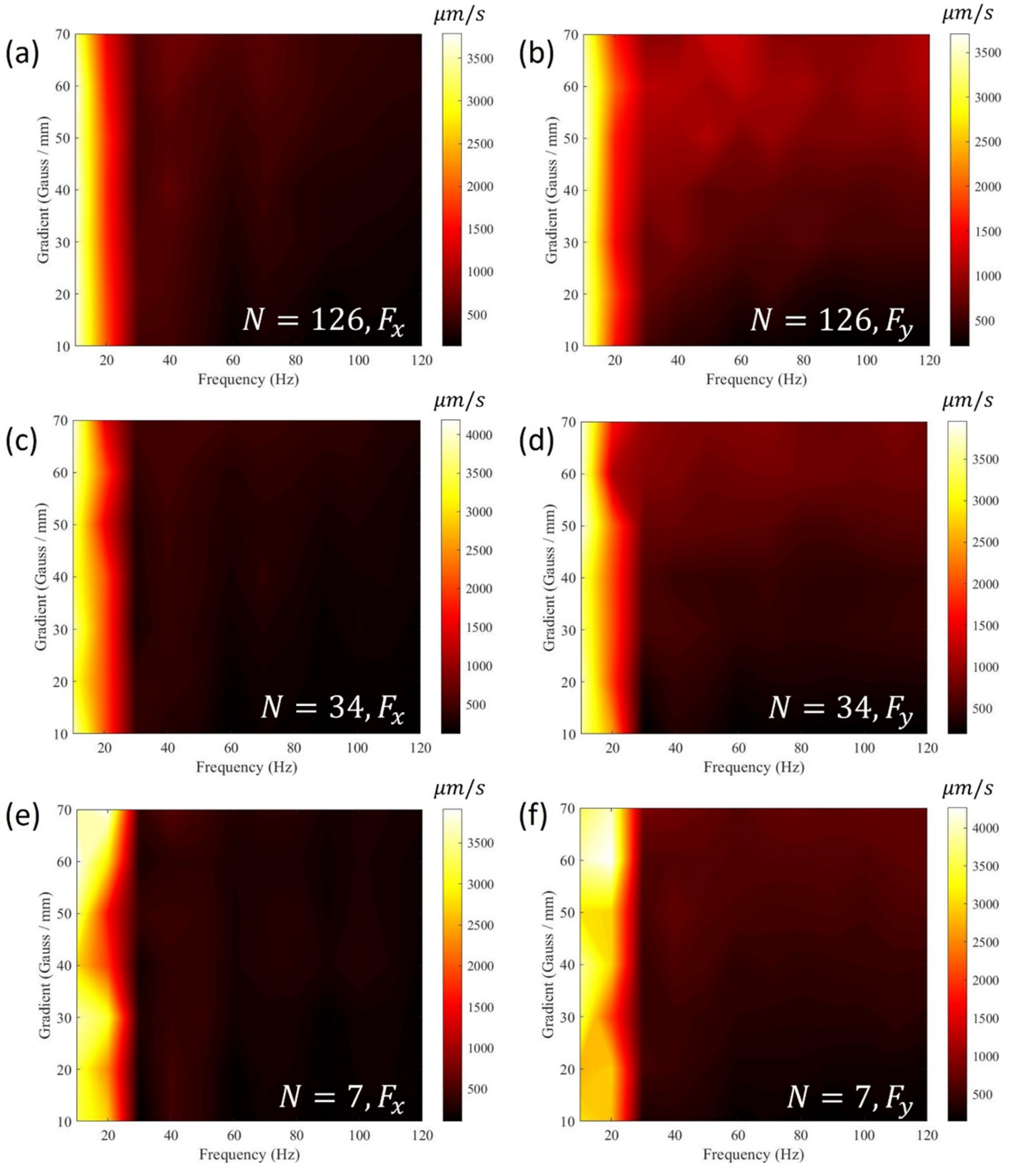

Supplementary Fig. 8. Locomotion speeds across various gradients and frequencies for chains aligned along the *y* direction. (**a**) Results for when there are 126 micro-disks and a gradient is acting on them in the (**i**) *x* direction and (**ii**) *y* direction. (**b**) 34 micro-disks. (**c**) Seven micro-disks. The error bars represent standard deviation over 5 s.

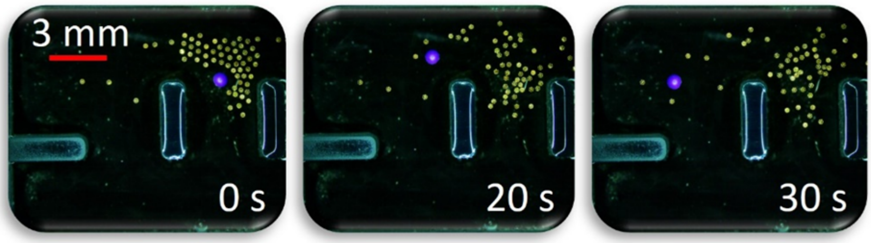

Fig. S9. Flow-induced collective object manipulation in the rotation mode. Video snapshots of the collective in the rotation mode inducing flow that propels a ball in the left direction.

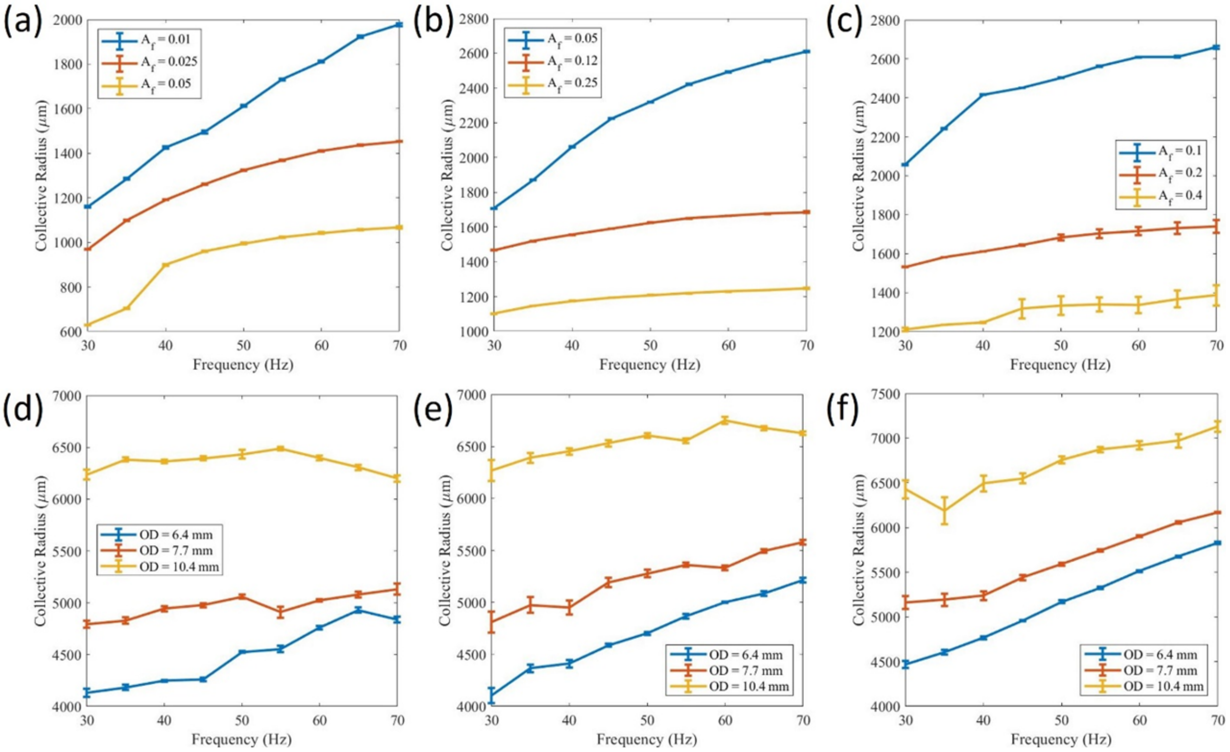

Supplementary Fig. 10. Collective radius when the micro-disks are within ring-like structures packed at various area fractions. (**a**-**c**): (a) 7 micro-disks, (b) 34 micro-disks, (c) 60 micro-disks. Collective radius when the micro-disks encapsulate ring-like structures with various OD (outer diameters) (**d**-**f**): (d) 34micro-disks, (e) 60 micro-disks, (f) 120 micro-disks. The error bars represent standard deviation over 10 s.

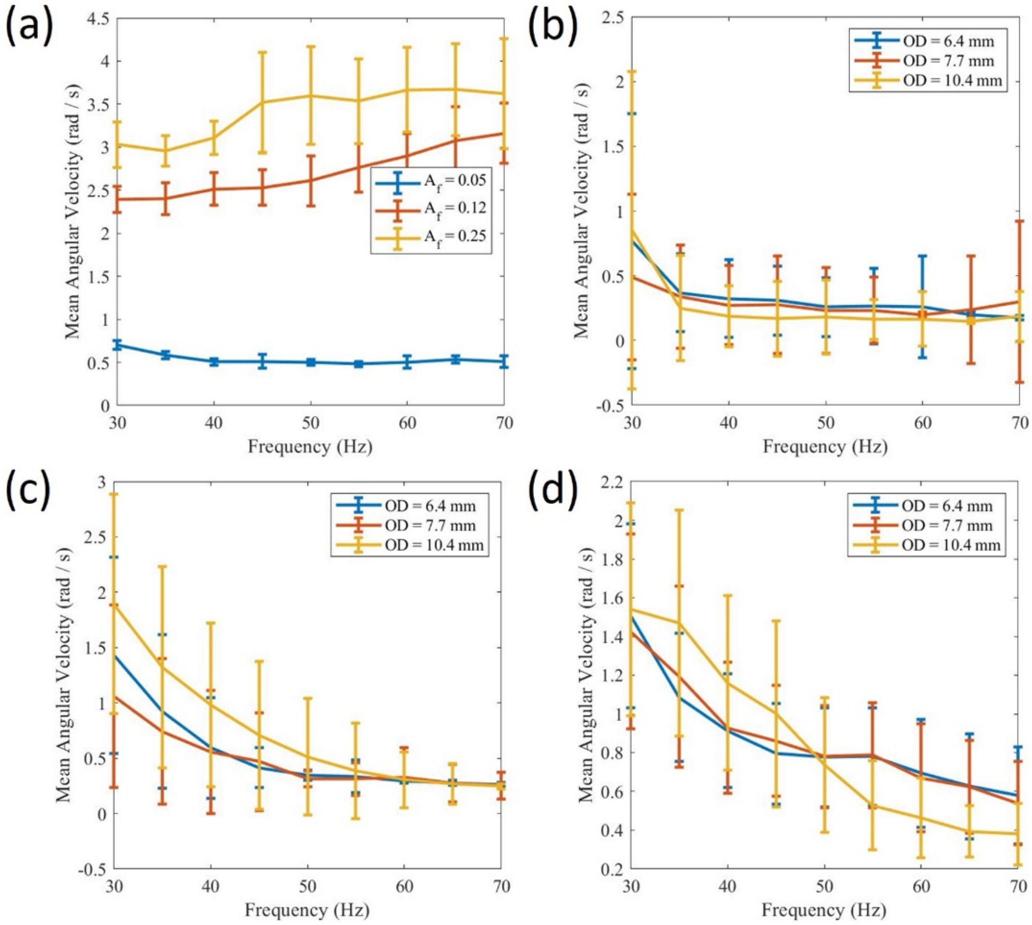

Supplementary Fig. 11. Mean angular velocity of micro-disks when they are within a ring-like structure. (**a**) 60 micro-disks. Mean angular velocity when the micro-disks encapsulate ring-like structures with various OD (outer diameters) (**b-d**): (b) 34 micro-disks, (c) 60 micro-disks, (d) 120 micro-disks. The error bars represent standard deviation over 10 s.

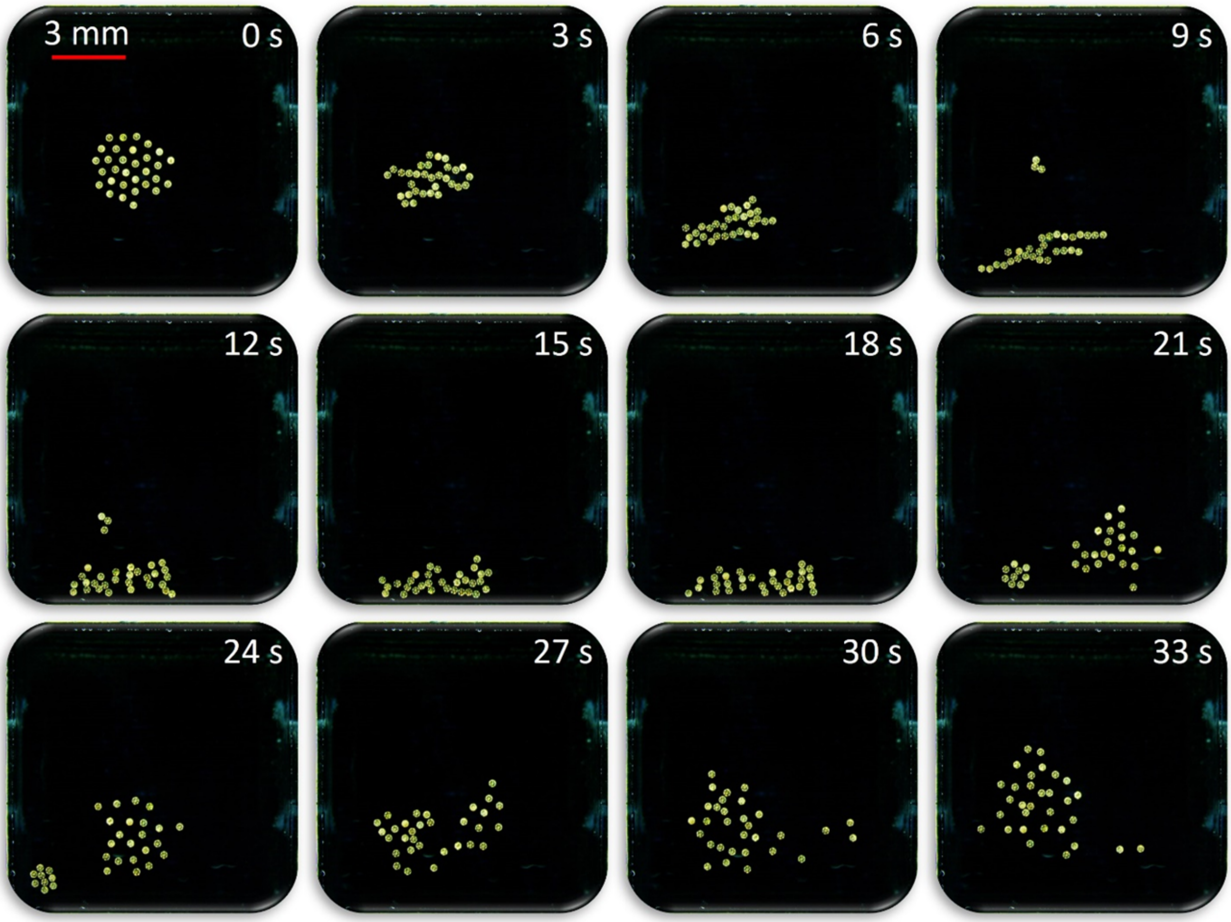

Supplementary Fig. 12. The collective separates into two clusters by using the arena boundary and then reforms a single cluster.
