# Supplementary material for: Microrobot collectives with reconfigurable morphologies, behaviors, and functions
Source: Nat Commun. 2022 Apr 26;13:2239. doi: 10.1038/s41467-022-29882-5 (PMC9043221; doi:10.1038/s41467-022-29882-5)
Supplement: Supplementary file 4 — Description of Additional Supplementary Information [file 41467_2022_29882_MOESM4_ESM.pdf]

Description of Additional Supplementary Information

The following are the legends for the supplementary Movie files:

Supplementary Movie 1. Overview of different collective formations: The behavior of a single micro-disk is shown when different magnetic field profiles are applied. Then the behavior of the collective is shown for the same conditions. The collective is shown when it is rotating, oscillating, static, in X and Y chain formations, and exhibiting the gas-like mode (GaSPP).

Supplementary Movie 2. First sequence of transitions between collective formations: The collective transitions through the following list of modes: rotation, static, oscillation, chain Y, chain X, oscillating chains, GaSPP, rotation, and static.

Supplementary Movie 3. Simulations of transitions between collective formations: The simulations showing the different collective behaviors in steady-state, like the ones shown in supplementary movie 1. Then the collective is simulated to replicate the order of mode transitions shown in supplementary movie 2.

Supplementary Movie 4. Second sequence of transitions between collective formations: The collective transitions through the following list of modes: rotation, chain X, static, chain Y, GaSPP, rotation, GaSPP, and oscillation.

Supplementary Movie 5. Third sequence of transitions between collective formations: The collective transitions through the following list of modes: GaSPP, chain Y, rotation, GaSPP, oscillation, chain X, GaSPP, and static.

Supplementary Movie 6. Coordinated locomotion of the collective in different formations: Four experiments are shown side by side where collectives exhibit rotation, static, oscillation, and chain modes and locomote in the Y direction when driven by magnetic field gradients.

Supplementary Movie 7. Magnetic field gradient control for following the “MPI” and “C” trajectories: Chains composed of seven micro-disks are driven by magnetic field gradients to trace out the MPI and C trajectories.

Supplementary Movie 8. Collective navigation through an intricate environment: The collective of 17 micro-disks switches between several modes and is driven by magnetic field gradients to locomote through narrow and open spaces.

Supplementary Movie 9. Contact-based collective object transport using the magnetic field gradients: The collective locomotes across an arena and forms Y chains to then push on a large object.

Supplementary Movie 10. Visualization of flow around the rotating collective: A dye is introduced at the left side of the rotating collective to visualize the local and global azimuthal flow fields.

Supplementary Movie 11. Flow-induced object transport using the rotating collective: The collective encapsulates a large object and then switches to rotation mode, thus generating flow fields that transport the object along the arena’s perimeter.

Supplementary Movie 12. Ring rotation from inside and outside: Rotating collectives are shown inside and outside of the ring structures to demonstrate the difference in direction of object rotation.

Supplementary Movie 13. Torque transfer between two rings: Adjacent ring structures are shown rotating about their common center of mass or in opposite directions when collectives are within each structure or inside only one of the structures, respectively.

Supplementary Movie 14. Rotation of various collective shapes: Collectives are shown rotating rod and star-shaped structures when they are within each structure.

Supplementary Movie 15. Comparison of dispersion using the rotation and GaSPP modes: The collective is shown to uniformly spread across a non-circular structure’s open area when it is rotating, and quickly but non-uniformly cover the structure’s open area when it is in the GaSPP mode.

Supplementary Movie 16. Collective splitting using the rotation mode: The collective lines up as chains along an arena’s boundary and then splits into two rotating clusters.

Supplementary Movie 17. Collective splitting using the GaSPP mode: The collective is shown dispersing from one side of an arena to another through the GaSPP mode, forming two rotating clusters, and then rejoining to the same side of the arena.
